# Levels of Satisfaction, Workload Stress and Support Amongst Informal Caregivers of Patients Receiving or Not Receiving Long-Term Home Nursing Care in Poland: A Cross-Sectional Study

**DOI:** 10.3390/ijerph16071189

**Published:** 2019-04-02

**Authors:** Zofia Stojak, Jacek Jamiolkowski, Slawomir Chlabicz, Ludmila Marcinowicz

**Affiliations:** 1 The Non-Public Health Care Centre OMNI-MED in Bialystok, 15-054 Bialystok, Poland; mmstojak@wp.pl; 2 Department of Population Medicine and Civilization Diseases Prevention, Medical University of Bialystok, 15-269 Bialystok, Poland; jacek909@wp.pl; 3 Department of Family Medicine, Medical University of Bialystok, 15-054 Bialystok, Poland; schlabicz@poczta.onet.pl; 4 Department of Primary Health Care, Medical University of Bialystok, 15-054 Bialystok, Poland

**Keywords:** informal caregiver, long-term care, nurse, COPE index

## Abstract

The role of informal caregivers was included in the Assumptions of the Long-Term Senior Policy in Poland for 2014–2020. The document acknowledged the necessity of diagnosing the needs of informal caregivers of elderly people and to implement systemic solutions that would enable the provision of assistance for them. In response, this study aimed to describe the situation of caregivers of patients receiving versus patients not receiving Long-Term Home Nursing Care (LTHNC; i.e., a formal program including regular visits by a nurse specializing in home care) in terms of caregiver socio-demographic characteristics, health self-assessment, work overload, satisfaction derived from being a caregiver, and the quality of perceived support. A cross-sectional study was conducted using the Carers of Older People in Europe (COPE) Index in 2015 in the north-eastern part of Poland involving 170 caregivers of patients supported with LTHNC and 86 caregivers of patients staying at home and not receiving LTHNC. We found that caregivers for patients receiving LTHNC were significantly less overloaded with care work than caregivers for patients without LTHNC support (*p* < 0.001). LTHNC support was also related to the level of satisfaction with providing care: Caregivers for patients receiving LTHNC were significantly more satisfied with performing their role and felt greater support than caregivers for patients without LTHNC (*p* < 0.001). Our study provides evidence for a positive relationship between LTHNC and the situation of informal caregivers of dependent elderly people at home. A formal program of visits by a nurse specializing in long-term home care may facilitate the provision by caregivers of better informal care to patients staying at home.

## 1. Introduction

As in many other European countries, Poland’s population is an aging one. According to the Central Statistical Office, at the end of 2016 the proportion of people aged 65 or older in the general population was 16.4%, whereas in 1990 it was 10.2% [1]. This highlights the growing need for various forms of support for dependent elderly people in their home environment. In addition, there is a need for more scientific studies to achieve a comprehensive understanding of the state of home care in Europe [2].

In Poland, long-term care at home for dependent elderly people is mostly provided by the closest family members (child, parent), with insufficient support from professional medical services. Apart from family caregivers, neighbors or friends sometimes provide care for a dependent person, acting as informal caregivers [3]. The role of informal caregivers was included in the Assumptions of the Long-Term Senior Policy in Poland for 2014–2020. The document acknowledged the necessity of diagnosing the needs of informal caregivers of elderly people and to implement systemic solutions that would enable the provision of assistance for them [4]. Long-Term Home Nursing Care (LTHNC) guaranteed by the Ministry of Health is the Polish government’s response to this need. As part of the national health insurance, LTHNC is a formal program to ensure the provision of extended nursing care at home to disabled elderly people (up to 40 points on the Barthel scale); the patient does not cover the expenses connected with this kind of care. Upon referral from a doctor, the nurse arranges the time of the initial visit to the patient and their caregiver. During that visit, the nurse assesses the patient using the Barthel scale and interviews the patient and the caregiver. The nurse then prepares the nursing diagnosis and the nursing plan, then discusses it with the patient and the caregiver [5]. The nurse visits the patient at least four times a week, and even more often in medically justified cases (including Saturdays, Sundays, and bank holidays).

According to the Regulation, LTHNC includes services provided by a nurse, such as nursing care following a nursing procedure; assistance in solving medical and social problems connected with independent functioning at home; assistance in obtaining rehabilitation equipment needed for proper nursing and rehabilitation at home; and preparing the patient for self-care and self-management. This form of care also involves supporting the family and preparing them to care for the dependent person [5]. Once a month, the nurse assesses the patient’s functional status using the Barthel scale. If the patient’s condition improves and the patient scores more than 40 points on the Barthel scale, they are removed from LTHNC for further supervision by a family nurse [6]. Thanks to LTHNC, dependent persons can stay in their home environment, avoiding institutional care. However, due to the high demand for professional nursing services at home and a limited number of contracted services of this kind, not all patients in Poland can receive LTHNC.

Literature review shows that the available studies mainly focused on the characteristics of patients who receive LTHNC, whilst little attention has been given to the caregivers [7]. A previous study carried out in Poland reported that the mean age of patients receiving LTHNC was 82 years old, and their mean functional status diagnosed with the Barthel scale was 17 points [8].

The aim of this work was to describe the situation of caregivers of patients receiving and not receiving LTHNC in terms of caregiver socio-demographic characteristics, health self-assessment, overload resulting from providing care, satisfaction from being a caregiver, and the quality of perceived support.

## 2. Materials and Methods

### 2.1. Design and Participants

The descriptive research was conducted at one point in time (a cross-sectional study design) [9]. The survey was carried out from August to December 2015 in Podlaskie Province (the north-eastern part of Poland), which has a population of approx. 1.2 million. The total number of disabled people receiving LTHNC in this region was about 700.

The study participants were the informal caregivers of patients of two institutions providing LTHNC in Podlaskie Province. The nurse invited all caregivers of patients of those two healthcare centers to take part in the study. She distributed 188 questionnaires and received back 170 properly filled-in questionnaires (response rate 90%). The respondents from the group not receiving LTHNC were selected by family nurses from three other healthcare centers in Podlaskie Province from amongst their patients. One hundred questionnaires were distributed amongst the informal caregivers, and 86 were returned (response rate 86%).

In both groups, the caregivers of patients aged 65 or older, staying at home, and who scored between 0 and 40 points on the Barthel scale were included in the research. These were chronically ill patients but without hospitalization needs, whereas they still required regular nursing care at home because of their essential health issues.

The Barthel Index is a short rating scale which measures functional independence in personal care and mobility (feeding, moving from wheelchair to bed and back, personal toilet, getting on and off the toilet, bathing oneself, walking on a level surface, ascending and descending stairs, dressing, controlling the bowels and controlling the bladder). It is used to monitor the performance of chronic patients and to indicate the amount of nursing care needed. The score range is from 0 to 100; higher scores indicate greater independence [10].

The patients who received LTHNC were cared for by a nurse who visited them at least four times a week for at least 60 min at a time. One LTHNC nurse cared for no more than 6 patients. If the patient’s condition improved and the nurse evaluated their functional status as over 40 points in the Barthel scale, then the patient was under the care of a family nurse. The patients received LTHNC for an average of 148 days (the shortest, 2 days; the longest, 3 years).

The patients who did not receive LTHNC were only under the care of a family nurse who may care for up to 2500 patients. Family nurses, like family doctors, care for both healthy and ill people who are on their patient lists. Home visits of a family nurse are only performed in medically justified cases. Taking into account the increasing number of chronically ill and disabled elderly people, this is insufficient in comparison to the real needs of these social groups.

Both LTHNC and services provided by a family nurse are financed by the National Health Fund on the basis of health insurance and are free for the patient. Patients who receive LTHNC and those who do not could both benefit from social care services, although patients in the former group would likely benefit from them to a greater extent because of the more efficient provisioning of services via the LTHNC nurse.

Ethics Approval and Consent to Participate: The study was approved by the Bioethics Committee of the Medical University of Bialystok, Poland. Resolution no. R-I-002/23/2013. All subjects gave their informed consent to participate in the study.

### 2.2. Instrument

The Carers of Older People in Europe (COPE) Index emphasizes the subjective assessment by a caregiver of his or her own situation and circumstances [11,12]. The COPE Index is used to evaluate the level of caregiving burden, the level of satisfaction with serving in this role, and the quality of support received. It is made up of three subscales: 1. Negative impact of care (NIoC); 2. Positive value of care (PVoC); and 3. Quality of support (QoS). There are four possible responses to the questions in the COPE Index: always = 4, often = 3, sometimes = 2, never or not applicable = 1.

The NIoC scale is assessed using the following questions: (1) Is caregiving too demanding? (2) Does caregiving cause difficulties in your relationships with friends? (3) Does caregiving have a negative effect on your physical health? (4) Does caregiving cause difficulties in your relationship with your family? (5) Does caregiving cause you financial difficulties? (6) Do you feel trapped in your role as a caregiver? (7) Does caregiving have a negative effect on your emotional well-being? In the NIoC scale, the caregiver could score from 6 to 24 points. The higher the score, the worse the caregiver’s situation. A score greater than 12 points was interpreted as the caregiver being overloaded.

The Positive Value of Care (PVoC) scale included the following questions: (1) Do you feel you cope well as a caregiver? (2) Do you find caregiving worthwhile? (3) Do you have a good relationship with the person you care for? (4) Do you feel that anyone appreciates you as a caregiver? In this scale the caregiver could score from 4 to 16 points. More points reflect a caregiver’s higher satisfaction. It was assumed that a caregiver who scored more than 12 points had greater satisfaction with caregiving. Scores between 4 and 12 points were interpreted to mean the caregiver did not feel satisfaction with their role.

The quality of support (QoS) scale was assessed based on the caregiver’s answers to the questions: (1) Do you feel well supported by your family? (2) Do you feel well supported by your friends and/or neighbors? (3) Do you feel well supported by health and social services? (4) Overall, do you feel well supported in your role as caregiver? In this subscale the caregiver could score from 4 to 16 points. More points reflected greater perceived support.

In addition, the following information about the caregivers was collected: Age, sex, self-assessment of health, the degree of relationship with the patient, employment status, and the distance from the patient’s place of residence. The data were collected by means of a family nurse (working in another health care structure and not engaged in LTHNC) directly contacting the patient’s caregiver. After learning the study procedures, family nurses qualified patients with 0–40 points on the Barthel scale for the study, and then carried out the survey amongst their caregivers.

### 2.3. Statistical Analysis

Pearson’s χ² test for independence was used in the statistical analysis to assess the relationships between qualitative variables. The Mann-Whitney test was used to compare the distributions of quantitative variables between two patient subgroups. Multivariate linear regression models were used to estimate the influence of LTHNC on COPE index subscales, adjusted for variables significantly related to receiving LTHNC. All calculations were performed using the IBM® SPSS® Statistics ver. 20.0 statistical package. Statistical hypotheses were verified at a significance level of 0.05.

## 3. Results

### 3.1. Caregiver Socio-Demographic and Health Status

The caregivers were mostly women, both in the patient group not receiving LTHNC (72.1%) and in the patient group receiving LTHNC (64.1%). The mean age of the caregiver for the LTHNC group was 56.69 (SD 11.9) years, and the mean age of the caregiver for the non-LTHNC group was 53.50 (SD 12.9) years; *p* = 0.006. In both groups, the role of the caregiver was most often performed by the patient’s child (46.5% in the non-LTHNC group and 38.8% in the LTHNC group); (chi^2^ = 1.552; *p* = 626). Caregivers were most often retired (36.1% of caregivers in the non-LTHNC group and 44.1% of caregivers in the LTHNC group). However, many of the caregivers worked full time: 30.2% and 28.8%, respectively (chi^2^ = 3.488; *p* = 0.322). The vast majority of caregivers in both groups lived together with the patient; however, the caregivers in the LTHNC group lived together with the patients significantly more often (74.7%) than the caregivers in the non-LTHNC group (62.8%) (chi^2^ = 3.914; *p* = 0.047). Caregivers from both groups mostly assessed their health status as good and very good (53.5% of caregivers in the non-LTHNC group and 46.5% of caregivers in the LTHNC group) or average (29.1% and 37.1%, respectively). The difference was not statistically significant (*p* = 0.434) (Table 1).

### 3.2. Mean Evaluation of the Negative Impact of Care, Positive Value of Care and the Quality of Support Amongst the Caregivers for Patients Receiving and Not Receiving LTHNC

**Figure 1:**
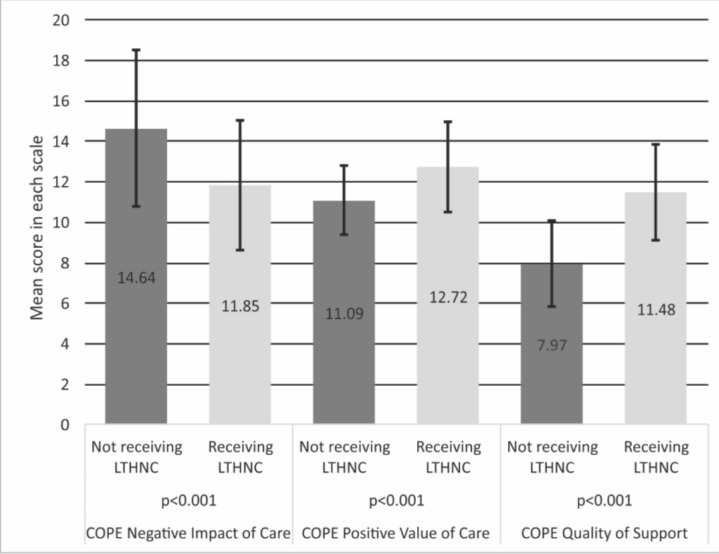
Mean evaluation of the Negative Impact of Care (NIoC), Positive Value of Care (PVoC) and the Quality of Support (QoS) depending on the presence of LTHNC (Carers of Older People in Europe (COPE) NIoC: Lower scores are better; COPE PVoC and COPE QoS: Higher scores are better).

Analyzing the mean values in the three subscales of the COPE Index, we found a positive correlation between LTHNC and the caregivers’ situation (Figure 1). The caregivers in the LTHNC group were significantly less overloaded by their care activities (11.85 points in NIoC; SD 3.2) than the caregivers in the non-LTHNC (14.64 points in NIoC; SD 3.9); (*p* < 0.001). LTHNC support was also related to the sense of satisfaction with providing care. The caregivers in the LTHNC group were significantly more satisfied with performing their role (12.72 points in PVoC; SD 2.2) than the caregivers in the non-LTHNC group (11.09 points in PVoC; SD 1.7); (*p* < 0.001). A statistically significant difference (*p* < 0.001) was also found between the two caregiver groups in terms of the perceived quality of support. The caregivers in the LTHNC group felt greater support (11.48 points in QoS; SD 2.3) than the caregivers in the non-group LTHNC (7.97 points in QoS; SD 2.2); (*p* < 0.001).

### 3.3. Relationship between Receiving LTHNC and COPE Index Subscales

Caregivers of patients receiving LTHNC score significantly lower in the Negative Impact of Care subscale than the caregivers in the non-LTHNC group. The older the caregiver, the higher the score in the Negative Impact of Care (*p* = 0.030). Dwelling together with the patient is related to a higher score in the Negative Impact of Care subscale (*p* = 0.001). Caregivers of patients receiving LTHNC score significantly higher in the Positive Value of Care subscale. The older the caregiver, the lower the score in the Positive Value of Care (*p* = 0.050). Dwelling together with the patient is not significantly correlated with the Positive Value of Care (*p* = 0.594). Caregivers of patients receiving LTHNC score significantly higher in the Quality of Support subscale. Neither the age of the caregiver nor dwelling together with the patient are significantly correlated with the Quality of Support (Table 2).

## 4. Discussion

Our study confirms that women comprise the majority of informal caregivers of dependent patients. The patient’s child (son or daughter) most often served as the caregiver. Spouses or partners also accounted for a significant proportion of caregivers. Another Polish study reported that elderly people prefer their family members to be the providers of their care needs [13]. In our study, the mean age of caregivers for patients receiving LTHNC was higher than that of caregivers in the non-LTHNC group. The older age of the caregivers in the LTHNC group was likely the reason why they wanted LTHNC for their dependent patients.

Family caregivers feel a greater burden of caregiving than formal caregivers, because they are emotionally engaged in the patient’s situation. In a cross-sectional study involving 328 family caregivers in Spain, the following typical profile of a caregiver was determined: woman around 60 years old, housewife, with primary education. Female sex, being a son or daughter of the patient, housewife employment status, and the care recipient being female were significantly associated with subjective strain on the caregiver [14]. A Finnish study, also using the COPE Index, showed that family caregiving was perceived as being very valuable, and the majority of family caregivers had a very close relationship with the person they were caring for, and were supported by the family [15].

In our study, we applied the COPE Index to gather information about the subjective feelings of caregivers, their relationships with their patients, and the problems of the quality of formal and informal support. The reliability and validity of this tool was tested amongst the caregivers of disabled people [16]. In the present study, by comparing the mean values of the three subscales of the COPE Index between the LTHNC and non-LTHNC groups of caregivers, we found statistically significant differences in favor of the former group. Regular visits by a nurse at the patient’s home—at least four times a week, and in justified cases, even more often (including holidays)—as well as the nurse’s role in preparing the caregiver to care for the patient, may have contributed to the sense of satisfaction with caregiving, reduced the feeling or actuality of overload, and improved attitudes to the usefulness of support.

LTHNC is an organizational solution in the long-term care system and is an example of so-called “good practice”, facilitating the provision of care for disabled people in their homes [17]. On the one hand, it ensures intensive nursing care in the patient’s home and, on the other hand, it is a kind of support for informal caregivers of disabled people. Moreover, it promotes the professional autonomy of a nurse and makes it possible to use the potential that nurses have.

Informal caregivers play an essential role in maintaining disabled elderly people at home as well as ensuring their support is comprehensive [18]. Taking into consideration the perspective of informal caregivers should be an integral part of the evaluation of functioning of home care. Support strategies for informal caregivers and promoting satisfaction from the provision of care are also important [19].

The strength of our study is that, in both groups, the data collection followed the same procedures, using a valid and reliable research tool tested under Polish conditions [20]. A limitation is the relatively low number of respondents (especially in the group not receiving LTHNC), which made a more detailed analysis of the findings impossible. Still, the sample was sufficient to achieve the research goals. Moreover, the study was only carried out in one region of Poland, which makes it impossible to generalize the findings.

## 5. Conclusions

Our study provides evidence for a positive relationship between LTHNC and the situation of informal caregivers of dependent elderly people staying at home. The caregivers for patients receiving LTHNC felt less stress and greater satisfaction in their role as caregivers, and they had the sense of receiving greater support than did the caregivers for patients not receiving LTHNC. Visits by a nurse providing long-term home care may facilitate the provision of care and help informal caregivers to provide better care. Greater availability of LTHNC may improve the quality of care for elderly and disabled people and the situation of their caregivers. A change in health policy and the extension of the LTHNC program are needed to improve long-term care for dependent elderly people staying at home. Repeating the study on a larger sample of people both receiving and not receiving LTHNC could enhance the reliability of the findings. Further research (especially qualitative) is necessary to provide in-depth data on the situation of caregivers and how they benefit from the nursing interventions.

## Figures and Tables

**Table 1:** Characteristics of caregivers of patients not receiving Long-Term Home Nursing Care (LTHNC) and receiving LTHNC.

	Not Receiving LTHNC		Receiving LTHNC	
Age	Mean (SD)		Mean (SD)	
	53.50 (12.9)		56.69 (11.9)	
*P*	0.006			
Sex	Not receiving LTHNC		Receiving LTHNC	
	n	%	n	%
Women	62	72.1	109	64.1
Man	24	27.9	61	35.9
*P*	0.181			
Self-assessment of health	Not receiving LTHNC		Receiving LTHNC	
	n	%	n	%
Good or very good	15	53.5	28	46.5
Average	25	29.1	63	37.1
Very poor or poor	46	17.4	79	16.5
*P*	0.434			
Relationship	Not receiving LTHNC		Receiving LTHNC	
	n	%	n	%
Daughter/Son	40	46.5	66	38.8
Spouse/Partner	17	19.8	40	23.5
Other family member	25	29.1	52	30.6
Friend/Neighbor	4	4.7	12	7.1
*P*	0.626			
Employment status	Not receiving LTHNC		Receiving LTHNC	
	n	%	n	%
Unemployed	14	16.3	29	17.1
Retired	31	36.1	75	44.1
Part-time	15	17.4	17	10.0
Full-time	26	30.2	49	28.8
*P*	0.322			
Place of residence	Not receiving LTHNC		Receiving LTHNC	
	n	%	n	%
Together with patient	54	62.8	127	74.7
Somewhere else	32	37.2	43	25.3
*P*	0.047			

**Table 2:** Relationship between receiving LTHNC and COPE index subscales (multivariate models adjusted for place of residence and age of caregiver).

Dependent Variable	Independent Variables	B, D	*P*	Adjusted R²
COPE Negative Impact of Care	Receiving LTHNC	−3.096	0.000	0.180
	Age of the caregiver	0.038	0.030	
	Dwelling together with the patient	1.534	0.001	
COPE Positive Value of Care	Receiving LTHNC	1.680	0.000	0.126
	Age of the caregiver	−0.021	0.050	
	Dwelling together with the patient	0.156	0.594	
COPE Quality of Support	Receiving LTHNC	3.536	0.000	0.347
	Age of the caregiver	−0.020	0.093	
	Dwelling together with the patient	0.327	0.311	
